# The Correlation Between Epiblepharon and Obesity in Pediatric Patients: A Retrospective Comparative Study

**DOI:** 10.3390/jcm15072506

**Published:** 2026-03-25

**Authors:** Hee Jin Yoon, Jung Hyo Ahn

**Affiliations:** 1 Department of Ophthalmology, Pusan National University Yangsan Hospital, Pusan National University School of Medicine, Yangsan 50612, Republic of Korea; gmlwls1447@naver.com; 2 Research Institute for Convergence of Biomedical Science and Technology, Pusan National University Yangsan Hospital, Yangsan 50612, Republic of Korea

**Keywords:** epiblepharon, body mass index, obesity

## Abstract

**Background/Objectives:** Epiblepharon is a common congenital eyelid anomaly in East Asian children, often associated with redundant skin and orbicularis oculi muscle overriding the eyelid margin. Recent studies have suggested that systemic factors such as body mass index (BMI) may contribute to its development. This study aimed to investigate the relationship between BMI and epiblepharon and to analyze the correlation between BMI and skin-fold height as a marker of eyelid structural redundancy. **Methods:** This retrospective comparative study included 100 pediatric patients (54 males, 46 females) aged 3–13 years who underwent surgical correction for lower eyelid epiblepharon and 100 age-matched controls without the condition. Preoperative height, weight, and skin-fold height were analyzed. Intergroup comparisons were performed using independent *t*-tests, and correlations between BMI and skin-fold height were evaluated using Spearman correlation. **Results:** There were no significant differences in overall BMI, obesity index, or prevalence of obesity defined as BMI ≥ 95th percentile between groups. Boys aged 7–8 years demonstrated significantly higher BMI in the epiblepharon group, and boys aged 9–10 years showed a significantly higher obesity index in the epiblepharon group, whereas boys aged 3–4 years showed significantly lower BMI. No significant differences were observed in girls. BMI was not independently associated with epiblepharon in multivariate logistic regression analysis (OR 1.06, 95% CI 0.96–1.16, *p* = 0.278). Among patients with epiblepharon, BMI showed a significant negative correlation with skin-fold height (r = −0.410, *p* < 0.001), suggesting increased orbicularis muscle redundancy in obese children. **Conclusions:** BMI was not independently associated with the presence of epiblepharon; however, age-specific differences were observed in certain male subgroups. Higher BMI was correlated with lower skin-fold height among affected patients, suggesting that adiposity may influence eyelid morphology in specific developmental stages. Further longitudinal studies are warranted to clarify the age-dependent relationship between obesity and epiblepharon.

## 1. Introduction

Epiblepharon is a congenital eyelid anomaly characterized by a redundant horizontal skin fold and overriding of the orbicularis oculi muscle, causing the eyelashes to be directed upward or inward toward the cornea, resulting in irritation of the cornea and conjunctiva [1,2]. This condition is commonly observed in East Asian children, including those in Korea, and typically involves the lower eyelids, particularly the medial portion.

Clinically, children with epiblepharon may exhibit symptoms including frequent eye rubbing, photophobia, tearing, and discharge [3]. However, many cases are identified incidentally during routine examination, as both patients and parents may be unaware of any notable symptoms.

Epiblepharon often resolves spontaneously as facial bone growth progresses, and with reported incidence declining from approximately 46% in infants under 1 year of age to approximately 2% among adolescents aged 13–18 years [4]. Due to this potential for spontaneous resolution, conservative management is typically attempted first for corneal irritation or epithelial erosion, with surgical correction typically required in cases with persistent symptoms or significant corneal erosions despite conservative therapy.

The etiology of epiblepharon is multifactorial, and the underlying mechanisms have yet to be fully elucidated. Proposed anatomical factors include overriding of the skin and orbicularis oculi muscle, lower insertion of the orbital septum, and a relative lack of vertical support for the pretarsal skin [5,6]. In epiblepharon, the eyelid margin remains in the normal position without tarsal plate inversion, distinguishing it from entropion.

Recently, systemic factors influencing periocular soft tissue volume have been proposed as potential contributors to the development and persistence of epiblepharon. In particular, body mass index (BMI), a widely used indicator of obesity, has been associated with increased subcutaneous and orbital fat volume, which may alter eyelid contour and exert inward pressure on the eyelid margin [7,8,9,10]. Given the global rise in childhood obesity, understanding the relationship between BMI and eyelid malpositions has become increasingly clinically relevant.

From a clinical perspective, clarifying the association between obesity and epiblepharon is particularly meaningful, as obesity represents a potentially modifiable systemic factor. If increased body mass contributes to the persistence or severity of epiblepharon in certain pediatric populations, lifestyle modification and weight control could potentially influence or reduce the need for surgical intervention. This consideration is especially important in pediatric patients, given that epiblepharon often improves spontaneously with growth and that avoiding unnecessary surgery during early childhood is a key clinical goal.

Despite this proposed association and its potential clinical implications, relatively few studies have systematically examined the relationship between BMI and epiblepharon. Therefore, in this study, we aimed to compare BMI between pediatric patients with epiblepharon and age-matched controls and to further investigate the correlation between BMI and skin-fold height as an indicator of eyelid structural redundancy. This study aims to provide a more comprehensive understanding of the potential role of obesity in the development and persistence of epiblepharon and to highlight its clinical relevance in the evaluation and management of affected children.

## 2. Materials and Methods

### 2.1. Study Design and Data Source

This retrospective comparative study was conducted at Pusan National University Yangsan Hospital, Republic of Korea. The medical records of 100 patients aged 3–13 years who visited the Pediatric Ophthalmologic Clinic at Pusan National University Yangsan Hospital between January 2018 and September 2019 were reviewed. Because this study was conducted as a retrospective analysis of existing medical records, it was granted IRB exemption. Institutional Review Board approval was obtained (IRB no. 55-2026-022, issued on 26 January 2026), and the study was conducted adhering to the Declaration of Helsinki.

### 2.2. Patient Identification

Epiblepharon is defined as an extra fold of skin pushing the eyelashes upward while maintaining a normal eyelid margin. The study includes one hundred patients (54 boys, 46 girls) who underwent surgical correction and were assigned to the epiblepharon group, with a control group consisting of 100 age-matched children (42 boys, 58 girls) without epiblepharon, recruited from pediatric ophthalmology clinics over the same period. Uncooperative patients, those who had incomplete medical records, or individuals with other underlying diseases (e.g., trichiasis or entropion) unrelated to epiblepharon were excluded.

Patients were stratified into age subgroups (3–4, 5–6, 7–8, 9–10, and 11–13 years) and analyzed separately by sex to evaluate age- and sex-dependent differences in BMI and eyelid morphology.

All patients underwent standard cilia rotation suture techniques. A crescentic resection of the skin and orbicularis muscle was performed 1–2 mm below the lower eyelid margin, followed by three to four non-absorbable rotational sutures between the pretarsal orbicularis oculi muscle and the tarsus. The skin was closed using continuous sutures of an absorbable material [11].

Lower eyelid skin-fold height was assessed using standardized preoperative frontal photographs obtained in primary gaze. Measurements were assessed according to Khwarg’s classification, and the severity of epiblepharon was determined by the position of the highest point of the skin fold. According to Khwarg’s classification system, patients whose skin fold was located below the lower eyelid margin were classified as Class I. Those with a skin fold positioned at or just below the lower eyelid margin were assigned to Class II. Patients in whom the skin fold extended above the eyelid margin and concealed less than one-third of the medial eyelid were categorized as Class III, whereas those whose skin fold lay above the eyelid margin and concealed more than one-third of the medial eyelid were classified as Class IV [12].

### 2.3. Body Mass Index (BMI) and Obesity

Obesity status was assessed using the obesity index and body mass index (BMI), as commonly applied by the Korea Disease Control and Prevention Agency (KDCA) National Health Information Portal. In children, because they are in a period of active growth, obesity was evaluated and compared according to sex- and age-specific standards.

The obesity index was calculated using the sex-, age-, and height-specific standard body weight (50th percentile). The formula used was as follows: obesity index (%) = (actual body weight–standard body weight for height) × 100/standard body weight for height. The standard body weight for height was obtained from reference data provided by the KDCA National Health Information Portal. An obesity index of 20–30% was classified as mild obesity, 30–50% as moderate obesity, and ≥50% as severe obesity [13].

Body mass index (BMI) was calculated as weight in kilograms divided by height in meters squared (kg/m^2^) and rounded to one decimal place. Height and weight were obtained from medical records at the time of preoperative evaluation. According to the growth charts, obesity was defined as a BMI at or above the 95th percentile for age and sex, based on the BMI-for-age growth curves provided by the KDCA National Health Information Portal.

### 2.4. Statistical Analysis

All analyses were conducted by SPSS Statistics version 25.0 (IBM Corp., Armonk, NY, USA) software. For comparisons of BMI between the epiblepharon group and the control group, independent comparisons were conducted using a *t*-test for continuous variables. In small subgroup analyses, the non-parametric Mann–Whitney U test was applied to account for small sample sizes and potential deviations from normality. Categorical variables were compared using the chi-square test or Fisher’s exact test. To find correlations between BMI and skin-fold height, data were analyzed using Spearman correlation. To account for potential confounding effects of age and sex, multivariate logistic regression analysis was performed to evaluate whether BMI was independently associated with the presence of epiblepharon. The dependent variable was group status (epiblepharon vs. nonepiblepharon), and BMI was entered as a continuous independent variable. Age and sex were included as covariates in the model. Odds ratios (ORs) with 95% confidence intervals (CIs) were calculated. A *p*-value < 0.05 was considered statistically significant.

## 3. Results

### 3.1. Patient Characteristics

Of 100 children with epiblepharon, 54 were boys and 46 were girls. The mean age of children with epiblepharon was 6.38 ± 2.46 years. The 100 children who were assigned to the control group consisted of 42 boys and 58 girls. The mean age was comparable between the epiblepharon and control groups. There were no significant differences in age or sex distribution between groups. (*p* > 0.05) (Table 1). Also, there were no significant differences between BMI, obesity index, and obesity by pediatric BMI percentiles between the two groups.

When obesity was defined as an obesity index ≥ 20%, the prevalence of obesity was not significantly greater in the epiblepharon group compared to controls. In addition, when obesity was defined according to a BMI ≥ sex- and age-specific 95th percentile, the prevalence of obesity defined as BMI ≥ 95th percentile did not differ between groups.

### 3.2. Correlation Between BMI and Epiblepharon

Analysis of the correlation between the occurrence of epiblepharon and BMI demonstrated no significant correlation in girls (Table 2). However, boys with epiblepharon in the 7–8 age groups exhibited significantly higher BMI compared with their counterparts without epiblepharon (*p* < 0.05). Interestingly, boys aged 3–4 years with epiblepharon showed significantly lower BMI than age-matched controls (*p* < 0.05).

### 3.3. Correlation of Obesity and Epiblepharon

When stratified by age and sex, the association between obesity and epiblepharon differed between boys and girls. In boys aged 3–4 years, the obesity index was significantly lower in the epiblepharon group compared with controls (*p* = 0.011). In boys aged 9–10 years, the obesity index was significantly higher in the epiblepharon group (27.94 ± 28.60 vs. −2.73 ± 9.88, *p* = 0.047). In other age groups (5–6, 7–8, and 11–13 years), no statistically significant differences were observed. When all boys aged 3–13 years were analyzed together, the obesity index tended to be higher in the epiblepharon group (9.49 ± 19.53 vs. 3.00 ± 14.88), but this did not reach statistical significance (*p* = 0.068). In girls, no significant differences in the obesity index were observed between the epiblepharon and control groups across all age groups. The prevalence of obesity, defined as BMI ≥ 95th percentile, did not significantly differ between groups in any age or sex category (Table 3).

### 3.4. Correlation Between BMI and Skin-Fold Height

**Figure 1:**
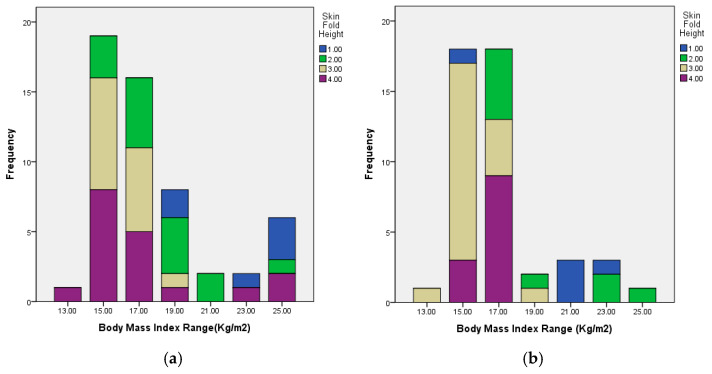
Distribution of skin-fold height classes according to BMI using a bar chart. (**a**) Male distribution. (**b**) Female distribution.

The skin-fold height classifications according to BMI categories are demonstrated in Table 4 and Figure 1. Among patients with epiblepharon, a higher BMI was significantly associated with a lower skin-fold height class (*p* < 0.05), suggesting that obese children tend to have less prominent skin folds and greater orbicularis muscle redundancy (Table 4). This correlation between BMI and skin-fold height was significant in both subgroups (*p* < 0.05) (Table 5).

### 3.5. Logistic Regression

In multivariate logistic regression analysis adjusted for age and sex, BMI was not independently associated with the presence of epiblepharon (OR 1.06, 95% CI 0.96–1.16, *p* = 0.278). Age (OR 1.01, 95% CI 0.89–1.15, *p* = 0.841) and sex (OR 1.56, 95% CI 0.88–2.74, *p* = 0.125) were also not significantly associated with epiblepharon (Table 6).

## 4. Discussion

Epiblepharon is a common congenital eyelid anomaly in East Asian children, predominantly affecting the lower eyelids. Although epiblepharon may remain asymptomatic in many cases, some patients experience foreign body sensations, photophobia, tearing, and discharge. In severe cases, corneal involvement such as keratopathy can occur, requiring surgical correction [3,4].

We investigated the relationship between BMI and epiblepharon by comparing children with moderate-to-severe disease who underwent surgical correction to age-matched controls without epiblepharon. This study demonstrated age and sex differences between BMI and epiblepharon in pediatric patients, with significantly higher BMI observed among boys aged 7–8 years with epiblepharon. This suggests that systemic factors may interact not only with genetic and anatomical factors but also with systemic conditions such as obesity at a specific age. These findings suggest that systemic factors influencing soft tissue volume may contribute to the persistence or severity of epiblepharon during specific stages of growth.

Interestingly, boys aged 3–4 years with epiblepharon demonstrated a significantly lower BMI than controls. This finding suggests that in early childhood, congenital anatomical factors—such as lower orbital septum insertion, immature midfacial skeletal development, and inherent pretarsal tissue configuration—may play a more dominant role than systemic adiposity. Because periorbital fat accumulation increases with age, the impact of obesity on eyelid morphology may become more apparent during mid-childhood rather than in early developmental stages. Therefore, the association between BMI and epiblepharon appears to be age-dependent.

Previous studies have reported similar associations between obesity and epiblepharon, although the affected age ranges varied [7,8,9,10]. Ahn et al. reported an association between obesity and epiblepharon in 223 girls aged 12–15 years diagnosed with epiblepharon [7]. Hayasaka et al. identified that Japanese children aged 6–11 years with epiblepharon had a significantly higher mean BMI than age-matched controls; in a consecutive study, 44% of children with a high BMI were diagnosed with epiblepharon [8,9]. Similarly, Yan et al. reported that Chinese children with epiblepharon had a higher mean BMI across all age groups than that noted in controls, although a statistically significant difference was only observed in boys aged 4–6 years [10]. Additionally, Takahashi et al. analyzed 509 patients with congenital lower eyelid epiblepharon and found that a higher BMI showed a trend toward increased risk of severe corneal involvement [14], suggesting that elevated BMI may not only be associated with the presence of epiblepharon but also with its clinical severity, although the association did not reach statistical significance in their logistic regression model. Furthermore, Wang et al. reported, in a large retrospective case–control study, that the median BMI was significantly higher in children with epiblepharon than in age- and sex-matched controls, and that overweight boys aged 4–9 years and adolescent girls aged 13–18 years had a significantly increased risk of epiblepharon, highlighting a sex-specific effect of obesity on disease risk [15]. Despite minor variations in age and sex distribution across studies, the prevailing view is that increased subcutaneous and orbicularis muscle tissues in the malar and lower eyelid regions can exert inward pressure on the eyelid margin, thereby contributing to the development or persistence of epiblepharon in children with a higher BMI.

In addition, given the relatively small subgroup size, this finding should be interpreted with caution. Further longitudinal studies are required to elucidate the age-dependent relationship between changes in BMI and eyelid morphology.

A key finding of this study is the significant negative correlation between BMI and skin-fold height. Higher BMI was associated with less prominent skin folds, which may reflect increased periorbital soft tissue volume rather than pure skin redundancy; however, direct anatomical measurements were not performed, and therefore, this interpretation remains speculative. The pathophysiology of epiblepharon in obese children may be related to soft tissue hypertrophy rather than to skin redundancy. Future studies using dynamic imaging modalities, such as ultrasound or magnetic resonance imaging, could further elucidate the soft tissue changes and anatomical mechanisms underlying epiblepharon in older children.

Clinically, these findings suggest that BMI should be considered in the evaluation and management of pediatric epiblepharon. High BMI may contribute to the persistence or recurrence of the condition, highlighting the importance of targeting the redundant orbicularis muscle during surgical correction rather than performing skin excision alone in obese children. Lifestyle modifications and weight management may also aid in reducing disease severity and recurrence risk in this population.

Significant differences in the obesity index were observed only in specific subgroups, particularly among boys aged 9–10 years, whereas BMI ≥ 95th percentile did not show significant differences in most age groups. These findings suggest that the association between obesity and epiblepharon may be age-dependent rather than uniformly present across childhood. Moreover, the obesity index (%), which reflects relative excess weight for height, may capture subtle adiposity-related effects that are not fully reflected by categorical BMI percentile cutoffs.

Because obesity and eyelid anatomy in children may both be influenced by developmental factors, we additionally performed multivariate logistic regression analysis, adjusting for age and sex. After controlling for these potential confounders, obesity was not independently associated with the presence of epiblepharon. This finding suggests that its influence may be intertwined with age-related anatomical development and sex-related structural characteristics of the lower eyelid. Larger prospective studies are warranted to further clarify whether obesity contributes directly to eyelid morphology or acts indirectly through growth-related mechanisms.

This study has several limitations. First, this study was retrospective, which limits the ability to infer causality. Although associations between BMI and epiblepharon were observed in specific age groups, the temporal relationship between changes in body composition and eyelid morphology could not be determined. Therefore, our findings should be interpreted as associative rather than causal. Second, BMI was used as an indirect surrogate marker of obesity. While BMI is widely accepted in epidemiological studies, it does not differentiate between fat and lean mass and provides no information regarding regional fat distribution. In particular, BMI cannot directly quantify periorbital fat volume or orbicularis muscle hypertrophy, which are hypothesized to influence eyelid morphology. Although we supplemented our analysis using obesity index and age- and sex-specific BMI percentiles based on national growth standards to reduce developmental bias, the absence of direct adiposity measurements—such as skin-fold thickness at other anatomical sites, bioelectrical impedance analysis, or orbital imaging—limits the mechanistic interpretation of our results. Third, stratification by age and sex resulted in small and uneven subgroup sizes in certain categories, particularly in the older age groups. Therefore, the significant associations observed in narrow age ranges should be interpreted cautiously and considered exploratory. Larger multicenter studies are required to validate these age-dependent findings. Fourth, the epiblepharon cohort consisted exclusively of surgically treated patients, representing moderate-to-severe cases. Consequently, the results may not be generalizable to mild or conservatively managed epiblepharon. This selection bias may have influenced the observed association between BMI and disease presence or severity. Although selection bias is an important limitation, it should be noted that epiblepharon frequently improves spontaneously with facial growth, and many cases can be managed conservatively. Therefore, our findings may reflect an association between BMI and clinically significant or persistent disease rather than simple disease presence. Future longitudinal studies are warranted to determine whether BMI influences the natural course of epiblepharon and whether weight control may reduce the likelihood of requiring surgical correction in selected pediatric patients.

## Figures and Tables

**Table 1:** Demographics and characteristics of patients.

	Epiblepharon	Non-Epiblepharon	*p*-Value
Patients	100	100	
Male, n (%)	54 (54.0%)	42 (42.0%)	0.119
Female, n (%)	46 (46.0%)	58 (58.0%)	
Age (years)	6.38 ± 2.46	6.05 ± 2.60	0.358
Height (cm)	119.80 ± 17.65	118.33 ± 18.13	0.562
Weight (kg)	27.09 ± 14.11	24.80 ± 9.91	0.185
BMI (kg/m^2^)	17.77 ± 3.76	17.08 ± 2.80	0.143
Obesity index (%)	7.06 ± 16.63	3.55 ± 15.81	0.128
Obesity index categories, n (%)			0.767
-Non-obese (<20%)	85 (85.0%)	88 (88.0%)	
-Mild (20–30%)	6 (6.0%)	5 (5.0%)	
-Moderate (30–50%)	6 (6.0%)	6 (6.0%)	
-Severe (≥50%)	3 (3.0%)	1 (1.0%)	
Obesity by pediatric percentiles, n (%)	11 (11.0%)	11 (11.0%)	1.000

**Table 2:** Comparison of BMI according to Age range and sex in epiblepharon and non-epiblepharon groups.

Age Range (Years)	Sex	Epiblepharon		Non-Epiblepharon		*p*-Value
		No. of Patients	BMI (Kg/m^2^)	No. of Patients	BMI (Kg/m^2^)	
3~4	Male	11	15.48 ± 0.64	15	17.17 ± 1.55	0.011 ^1,†^
	Female	14	16.17 ± 1.07	16	15.63 ± 1.77	0.200 ^†^
5~6	Male	16	16.92 ± 2.10	11	16.66 ± 0.27	0.802 ^†^
	Female	15	16.78 ± 2.01	19	16.14 ± 1.88	0.399 ^†^
7~8	Male	15	18.96 ± 2.98	6	17.37 ± 3.66	0.010 ^1,†^
	Female	10	17.74 ± 3.12	11	18.44 ± 3.86	0.696 ^†^
9~10	Male	6	24.76 ± 5.14	5	17.94 ± 1.39	0.329 *
	Female	6	19.16 ± 3.16	11	19.31 ± 3.46	0.956 ^†^
11~13	Male	6	24.10 ± 8.77	5	18.22 ± 2.96	0.329 *
	Female	1	21.02	1	15.50	-
3~13	Male	54	18.25 ± 4.57	42	17.24 ± 2.38	0.196 ^†^
	Female	46	17.20 ± 2.44	58	16.96 ± 3.09	0.664 ^†^

^1^ A *p*-value < 0.05 was considered statistically significant; * comparison between groups by Mann–Witney test; ^†^ comparison between groups by *t*-test.

**Table 3:** Comparison of the obesity index and obesity according to pediatric percentile in subgroups of age range and sex in the epiblepharon and non-epiblepharon groups.

Age Range (Years)	Sex	Obesity Index			Obesity (BMI ≥ 95th), n (%)		
		Epiblepharon	Non Epiblepharon	*p* Value	Epiblepharon	Non Epiblepharon	*p*-Value
3~4	Male	−1.43 ± 4.15	6.92 ± 10.83	0.011 ^1^	0 (0.0%)	2 (12.5%)	0.499
	Female	1.91 ± 7.33	−1.72 ± 15.71	0.405	0 (0.0%)	2 (11.8%)	0.488
5~6	Male	6.56 ± 11.65	4.27 ± 16.50	0.706	1 (6.2%)	1 (10.0%)	1.000
	Female	6.63 ± 12.75	3.91 ± 11.66	0.530	2 (13.3%)	0 (0.0%)	0.199
7~8	Male	10.42 ± 15.76	4.08 ± 22.37	0.546	1 (6.7%)	1 (16.7%)	0.500
	Female	4.67 ± 16.00	9.53 ± 20.69	0.564	0 (0.0%)	2 (20.0%)	0.474
9~10	Male	27.94 ± 28.60	−2.73 ± 9.88	0.047 ^1^	2 (33.3%)	0 (0.0%)	0.455
	Female	3.09 ± 14.42	8.64 ± 20.44	0.526	1 (16.7%)	1 (9.1%)	1.000
11~13	Male	16.53 ± 36.42	−7.65 ± 15.59	0.184	3 (50.0%)	0 (0.0%)	0.182
	Female	2.38	−10.89	-	0 (0.0%)	1 (100%)	-
3~13	Male	9.49 ± 19.53	3.00 ± 14.88	0.068	7 (13.0%)	4 (9.5%)	0.751
	Female	4.22 ± 12.01	3.95 ± 16.56	0.926	4 (8.7%)	7 (12.1%)	0.751

^1^ A *p*-value < 0.05 was considered statistically significant.

**Table 4:** Comparison of skin-fold height according to BMI range in epiblepharon.

BMI Range (Kg/m^2^)	Male Epiblepharon (Number of Class 1:2:3:4)	Female Epiblepharon (Number of Class 1:2:3:4)
<14	0:0:0:1	0:0:1:0
14–15	0:3:8:8	1:0:14:3
16–17	0:5:6:5	0:5:4:9
18–19	2:4:0:0	0:1:1:0
20–21	0:2:0:0	3:0:0:0
22–23	1:0:0:1	1:2:0:0
>24	3:1:0:2	0:1:0:0

**Table 5:** Correlation of skin-fold height and BMI range in epiblepharon.

	Number (N)	Correlation Coefficients (*R*)	*p*-Value
Male Epiblepharon	54	−0.366	0.006
Female Epiblepharon	46	−0.569	0.000
Total Epiblepharon	100	−0.410	0.000

**Table 6:** Factors influencing epiblepharon.

Variable	OR	95% CI	*p*-Value
BMI	1.06	0.96–1.16	0.278
Age	1.01	0.89–1.15	0.841
Sex	1.56	0.88–2.74	0.125

## Data Availability

The data presented in this study are available upon reasonable request from the corresponding author. The data are not publicly available due to privacy and ethical restrictions.

## Abbreviations

BMI	Body Mass Index
